# The Collective Voice of Medicine

**Published:** 1921-03-19

**Authors:** 


### THE COLLECTIVE VOICE OF MEDICINE.

Under this title the hospital public may have noticed a number of references to the outstanding feature of present-day medico-politics. But it is doubtful if the situation has been made even approximately clear to them. Not only medical men but every class of health worker in the country are intimately concerned in this matter. The Government, in its proposed reconstruction of health services, must have available representative professional opinion of all types. The urgent problem of the moment is, how shall Dr. Addison's demand for this collective voice be met? Obviously there must be developed some mechanism whereby all the various professional bodies can unite and have their views satisfactorily represented. Above all, it must be realised it is not medical opinion alone which is needed in administrative medicine.

The most remarkable event in this direction is the somewhat ponderous formality by which the British Medical Association invites its members to adopt articles and by-laws placing the Association in a position to become in part a Federation. The idea of a federation under the ægis of which all manner of medical and allied bodies can co-operate is excellent, but, in view of the great difficulty of getting even purely medical opinion into one representative channel, it appears to be particularly unfortunate that a single medical body should attempt itself to become this federation. The more striking is this, and the more obvious that the B.M.A. were better occupied in devoting its leadership to the doctors themselves, when it is realised that a Federation in every way adapted for complete representative functions is already in existence.

The Federation of Allied and Medical Societies owes its formation to a widespread realisation that the "collective voice" needed is that which can throw light on the broadest principles of health administration. In this sense, the medical voice or a predominant medical voice is not sufficient. Dr. Addison himself moved the first resolution at the Federation's inauguration, and as recently as last month he wrote expressing his gratification at the work it has already accomplished. He has, indeed, gone further, and, realising the inherent individualism of professional bodies and hoping for agreement between them, he has written to the Royal Colleges and to the B.M.A. itself, suggesting co-operation.

In our view no one existing body can ever represent the whole professional outlook, and although one is now attempting to do so, the disadvantage remains that this one still tends to be predominant. On the other hand, the Federation, acting merely as a federating *force*, includes all the collateral professions and need suffer from no rivalries.

In view of the antagonism which appears to be inevitably developing between the already established Federation and the proposers of the new, it may not be out of place to state that no Society or other body which has become a member of the Federation of Allied and Medical Societies has subsequently withdrawn. The present Council is now about to appoint others to control and superintend the work of the various branches of the Federation, as it was always intended should be done when the growth of the Federation necessitated such division. A medical council, an allied professions council, and a citizens' council are proposed, the various bodies in the Federation being represented on that council appropriate to the professions they represent. It is hoped shortly to move into more spacious quarters, where a Conference Hall will be provided free, with other services, from the constituent bodies. With the *Lancet* we can certainly agree, that "the Federation seems likely to make good its claim to guide public opinion without interference in the internal affairs of its constituents. Those who prefer substance to formality can hardly fail to wish it well."

